# Circadian temperature rhythm in breeding sows: differences between days in oestrus and anoestrus after weaning

**DOI:** 10.1186/s40813-024-00369-7

**Published:** 2024-05-21

**Authors:** P. Sánchez-Giménez, A. Martínez-Nicolas, J. A. Madrid, R. Fernández, L. Martínez-Alarcón, F. Murciano, A. Muñoz, G. Ramis

**Affiliations:** 1 Agropor SL, Las Torres de Cotillas, Murcia, Spain; 2 Departamento de Fisiología, Universidad de Murcia, Murcia, Spain; 3 Instituto Murciano de Investigación en Biomedicina (IMIB), Murcia, Spain; 4 UDICA, Hospital Clínico Universitario Virgen de La Arrixaca, Murcia, Spain; 5 Departamento de Producción Animal, Facultad de Veterinaria, Universidad de Murcia, Murcia, Spain

**Keywords:** Cyrcadian rithm, Body temperature, Anoestrus, Cosinor

## Abstract

**Background:**

Mammals are subject to circadian rhythms for the control of various physiological events. One of the parameters known to be subject to variations throughout the day is body temperature, which is also subject to influences such as environmental temperature. However, there are not many studies on these rhythms in breeding sows. The aim of this study was to determine the circadian parameters for body temperature in post-weaning sows during oestrus period, throughout the seasons in a warm climate.

**Results:**

Differences were observed in inter-daily stability, intra-daily fragmentation and cycle length comparing the summer sows with the other seasons. Differences were also observed in the period that the sows were in oestrus compared to the non-oestrus period for intra-daily fragmentation, with these differences being more important in the warm seasons compared to the cold seasons. The parameters normalised by COSINOR also showed significant differences when comparing seasons, especially in the acrophase of the temperature maximum. Another significant finding was an increase in vaginal temperature during oestrus in sows monitored in summer compared to the other seasons. Correlations between body, vaginal and environmental temperature were observed.

**Conclusion:**

There is a seasonal influence on the circadian rhythm of temperature and summer is clearly the season with the greatest differences in circadian parameters when compared to the other seasons. The extreme summer conditions seem to definitely influence this rhythm and make the body and vaginal temperature of the sows different from the rest of the year. The increase in period robustness in both body and vaginal temperature during the days when sows are in oestrus could be related to the hormonal events of oestrus and ovulation and seems to be independent of weather since it occurs in all controlled seasons. However, this robustness is significantly higher in summer than in the other seasons both in the oestrus period and on days when sows are not in oestrus.

## Introduction

In physiology, body temperature was one of the first parameters to be monitored in order to determine a circadian rhythm, as early as the nineteenth century (Chossat, 1843 and Davy, 1845, cited by Refinetti [1]). Research in this field has mainly been conducted in humans, where the circadian temperature rhythm has been linked to metabolism [1], aging and physical activity [2], thermoregulatory capacity [3] or reproduction [4, 5]. Another important point is the acquisition of temperatures, both core and skin, since the first approaches with classical thermometry (rectal or vaginal), have moved on to the use of non-contact elements such as infrared thermography [6–10], or the use of continuous recording devices such as Thermocron [11], bio-loggers [12] or encapsulated radiotransmitters [13] implanted subcutaneously, intraperitoneally or intravaginally [14].

However, although the first studies of circadian temperature variation in pigs were started almost 50 years ago[15], there is not abundant literature on circadian temperature rhythms in farm animals, and we only found some references in cattle [12, 13, 16], rabbits [17] or pigs [10]. Among them, the main interest of continuous or discontinuous temperature control in breeding sows is focused on the detection of oestrus, as fertility and prolificacy will depend on the efficiency in detecting the onset of oestrus, and consequently, on the timing of seminal doses. This is especially important in intensive production where, in countries such as Spain, artificial insemination-based breeding encompasses more than 97% of breeding sows. For the same reason, determining the time of ovulation is another point of interest related to the acquisition of body, rectal, vaginal, or skin temperature.

The objectives of this work were to describe the circadian rhythm of temperature in post-weaning sows and to differentiate the days when the sow was in oestrus and the days when she was not in oestrus, as well as its relationship with vaginal temperature during the same period. We also aimed to find out the relationship between circadian parameters, vaginal temperature, environmental clime parameteres and reproductive performance of farrow before and after controlling temperatures.

## Material and methods

### Animals and farm

All measurements were carried out at the Agropor farm (Agropor SLU, Torres de Cotillas, Spain), located in SE Spain (38°01′36″ N, 1°16′46″ W). The farm has 9600 sows, about 500 farrowings per week and specializes in the production of 6 kg piglets. Production is multi-site and the nursery is 1 km from the farm and the integrated finishing is distributed throughout the Region of Murcia.

The sows are hyperprolific Landrace × Large White, from two commercial genetics and there is weekly management. All breeding is based on artificial insemination, with semen purchased from an artificial insemination centre and administered by intracervical (40%) or postcervical (60%) insemination. The sows are fed by adjustable dosing units and the feeding regime is adjusted based on the body condition of the sow, subjectively assessed by a technician and corroborated by measuring backfat and loin depth by wireless ultrasound.

When the measurements were carried out, the sows were weaned at an average of 24 ± 1.2 days of lactation and immediately after weaning were housed in individual cages in day-lit sheds with environmental control based on natural ventilation managed by the animal handlers at each time of the day. All batches were measured in the same building to avoid possible variations induced by the location of the building within the farm.

The sows were randomly selected, and there was no difference in parity comparing the four seasons controlled (Winter = 2.36 ± 0.46, spring = 3.74 ± 0.41, summer = 3.96 ± 0.35 and autumm = 3.5 ± 1.23; *p* = 0.1), but in five batches, as the spring was sampled in March and June (EARLY SPRING and LATE SPRING), in order to have both climatic extremes of that season. The total study included 22 gilts, 10 s farrowing, 11 third, 10 fourth, 14 fifth, 6 sixth and seventh, 2 eighth and 1 ninth farrowing sows. This distribution reflects the usual census of the farm.

### Oestrus detection

Oestrus detection started the day after weaning, in the presence of a group of 6 boars. This was done twice a day (morning and afternoon) and took into consideration the presence of visible signs on the external genitalia (reddening and oedematisation of the vulva, presence of clear mucus) and the reproductive behaviour of the sows in the presence of the boars: erect ears and tail, nervousness, vocalizations and above all the immobilisation reflex, both in contact with the boars and with the farm technicians. The earliest time (morning or evening) when these signs will be seen is recorded as the onset of oestrus.

### Temperature recording

The temperature of 85 sows was recorded for seven days post-weaning over one year, distributed as follows: 6 sows in autumn, 17 in winter, 32 in spring (early spring n = 17 and late spring n = 15) and 29 in summer. Of this total, 10 sows were eliminated for not coming into oestrus during the first 7 days post-weaning and finally skin temperature data were obtained from 65 animals, as 10 dataloggers were lost or did not record data correctly. It was decided to sample sows at the beginning and end of spring as this is the season with the greatest variation in the Region of Murcia, and to ensure that we acquired all the information relating to this season.

Skin temperature recordings were carried out using Thermochron® i-buttons dattaloger DS1921H-F5 (Maxim Integrated Inc., USA), which had been previously validated for this task [11]. These devices are self-contained, do not require recharging and can store 2049 records, with a recording range of 14–46 °C. Once the temperatures were recorded, the information was downloaded by means of a Maxim Integrated One wire device and using the software one wire viewer® (Maxim Integrated, USA) downloading records as an excel® data sheet (Microsoft Inc, USA).

The Thermocron datalogger were fixed in the cervical area of the sows using a DS9093S wall mount nylon ring (Maxim Integrated, USA) and sutured to the skin by means of silk suture 2.0. The suture was applied using a local topic anaesthetic gel with lidocaine, 2.5% and prilocaine; 2.5% (ANESTOPIC, Spain), specifically formulated for the insertion of needles in the skin. Previously, the hair of the area was removed with depilatory cream. It was avoided the use of razor blades to prevent the skin irritation derived from the shaving. The skin was cleaned with alcohol and then the datalogger was sutured. The datalogger had been previously validated for this purpose comparing the value obtained on the skin with the temperature recorded in subcutaneous cervical area with the same device.

On the other hand, vaginal temperature was recorded by clinical veterinary thermometer inserted three times a day (7:00, 12:00 and 17:00 h) until such time as the sows did not show signs of oestrus. Records were differentiated depending on whether it was the period immediately prior to the onset of oestrus symptoms (Tvagbfr), during oestrus (Tvagoes) or after the cessation of oestrus symptoms (Tvagaft).

### Meteorological and environmental measurements

The ambient temperature and relative humidity of the environment was recorded by means of continuous recording thermohygrometers. In addition, the climatological data referring to temperature: average maximum (Tmax), average minimum (Tmin) and average (Tmean) temperature per day during the week studied, as well as the time at which Tmax (HoraTmax) and Tmin (HoraTmin) and the absolute Tmax and Tmin (Tmaxabs and Tminabs) occurred were obtained from the records of the Spanish Meteorological Agency (Agencia Española de Meterología; AEMET; using the OpenData portal AEMET, https://www.aemet.es/es/datos_abiertos/AEMET_OpenData accesed on March 24th, 2023) taking as reference the Alcantarilla Military Air Base located less than 8 km from the farm where the test was carried out (37°57′29″ N, 1°13′43″ W).

### Circadian temperature cycle values calculation

Parameters related to the circadian rhythm proposed by Witting et al. [18] were calculated: Interdaily stability (IS) which quantifies the similarity between the recorded 24 h cycles, Intradaily fragmentation (IV) which quantifies the fragmentation of the rhythm, the rhythm amplitude (AR) calculated as the difference of the temperature value obtained in period M8 and that obtained in period L8, the average temperature of the 8 consecutive hours with lower temperature °C (L8), the average temperature of the 8 consecutive hours with higher temperature in °C (M8), Middle moment of the L8 (TL8) in hours, Middle moment of the M8 (TM8) in hours and Mean as the average of all these data. These parameters were compared between the four climatic seasons and the days when sows were in heat versus days when sows were not in heat.

On the other hand, a standard cosinor analysis was performed as previously widely described [19–21], by means of the Cosinor v 3.1 programme (Reffineti, 2015) to calculate the period (time interval between phase reference points), robustness (repeatability of the cyclicity over the studied period), Mesor (rhythm-adjusted mean), Amplitude (half of the distance between the highest and lowest value within the period)and acrophase (timing of the cosine maximum) in each of the periods studied.

### Reproductive performances

Production parameters were recorded both immediately before (_B) and after (_A) the temperature data were recorded. The data recorded were: total piglets born (TBP), piglets born alive (PBA), stillborn piglets (SBP), piglets born mummified (MMP), piglets weaned (WP) and the wean-to-service interval (WSI).

### Statistics

The differences for the circadian temperature cycle parameters were assessed by Student’s T test with Levene’s test for variance equally, and the relation among parameters were assessed using partial correlations corrected for season and cycle of the sow.

Differences between circadian parameters during oestrus and non-oestrus days in sows were determined by a paired samples t-test.

## Results

### Climate data

The meteorological data provided by AEMET for each of the periods studied are shown in the following table (Table 1).

**Table 1:** Meteorological data during each of the studied periods

	Tmed (°)	Tmin (°)	Tmax (°)	Tmaxabs (°)	Tminabs (°)	HoraTmin (hh:mm)	HoraTmax (hh:mm)	ETD
Winter	9.42 ± 0.44^b^	2.42 ± 0.57^b^	16.45 ± 0.87^a^	17	− 0.20	6:10 ± 0:46	14:30 ± 11:09	0
Early spring	12.76 ± 0.52^a^	6.55 ± 1^c^	19.04 ± 0.96^a^	24	2.5	7:47 ± 2:33	12:43 ± 2:33	0
Late spring	23.14 ± 0.75^c^	14.86 ± 0.89^d^	31.37 ± 1^b^	35	11.20	4:41 ± 0:12	13:39 ± 0:31	5
Summer	28.69 ± 0.18^d^	20.9 ± 0.32 ^e^	36.49 ± 0.49^c^	39	20.3	4:35 ± 0:17	14:10 ± 0:13	7
Autumm	13.96 ± 0.39^a^	9.62 ± 0.92^a^	18.36 ± 0.62^a^	22	4.5	7:02 ± 2:39	14:09 ± 7:04	0
*P*-value	< 0.0001	< 0.0001	< 0.0001			NS	NS	

Where Tmed = average temperature over the period, Tmin = average of minimum temperature, Tmax = average of maximum temperature, Tmaxabs = highest value recorded over the entire period, Tminabs = lowest value recorded over the entire period, HoraTmax = hour for the Tmax record, HoraTmin = hour for the Tmin record, ETD = extreme temperature (if > 30 °C) days in number

Different superscripts in the same column indicate significant differences

As expected, there was a significant difference between all measurement times for Tmin, but no difference between autumn and early spring for Tmed and between autumn, winter and early spring for Tmax. There was also no difference in the time at which Tmax or Tmin occurred when comparing the five measurements. The lack of differences for Tmax except for summer, is an expression of the subtropical climate registered in SE Spain.

The data for environmental temperature inside the barn, relative humidity and brightness measured in the farm are given in Table 2.

**Table 2:** Environmental data recorded during the whole experience

Batch	T_ambDL_W	T_ambDL_O	HRA_W	HRA_O	Lum_W	Lum_O
Winter	15.55 ± 0.29^a^	15.86 ± 0.31^a^	58 ± 0.49^a^	63.92 ± 0.97^a^	85.89 ± 2.01^a^	90.48 ± 5.43^a^
Early spring	18.06 ± 0.06^b^	17.92 ± 0.09^b^	57.72 ± 0.26^a^	57.96 ± 0.73^b^	84.72 ± 7.44^a^	61.24 ± 9.48^a^
Late spring	27.18 ± 0.13^c^	26.61 ± 0.36^c^	49.09 ± 0.94^b^	51.18 ± 2.54^c^	143.61 ± 22.46^b^	110.13 ± 20.59^a^
Summer	31.82 ± 0.14^d^	32.09 ± 0.29^d^	39.52 ± 0.38^c^	37.41 ± 1.08^d^	399.03 ± 12.18^c^	440.32 ± 86.80^b^
Autumm	20.39 ± 0.22^b^	18.93 ± 0.29^b^	72.28 ± 0.10^d^	73.79 ± 0.46^e^	56.74 ± 4.23^a^	37.80 ± 1.52^a^
*p*-value	< 0.0001	< 0.0001	< 0.0001	< 0.0001	< 0.0001	< 0.0001

Where: T_ambDL_W = average temperature over the entire period recorded; T_ambDL_O = average temperature during the oestrus period; HRA_W = Relative ambient humidity during the entire period; HRA_O = Relative ambient humidity during oestrus period; Lum_W = luminosity during the entire period; Lum_O = luminosity during the oestrus period

Different superscripts in the same column indicate significant differences

There were differences in the mean temperature recorded inside the house comparing all measurements except autumn and early spring, although during the days of oestrus there was a difference between all batches. The relative humidity of the environment showed differences between all batches on the days when the sows were in oestrus, but in the total period there was no difference between winter and early spring. And with respect to brightness, there were differences between late spring and summer with the other periods, although in the days of oestrus only summer showed differences with the other periods.

There was no difference between the overall period and the days that sows showed oestrus within each of the batches.

Evidently there was a positive correlation between the climatic parameters collected by AEMET and those recorded in the building higher than r = 0.980 in all cases (*p* < 0.05), except between Tmax and HRA_W which, as expected, was negative (r = -0.893, *p* = 0.041).

### Oestrus detection and length

**Fig. 1:**
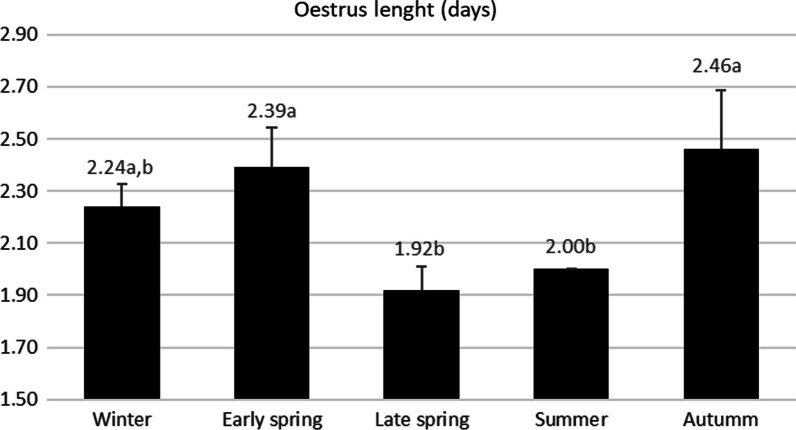
Length of oestrus sorted by batch studied

All oestrus were detected between 2nd and 4th days post-weaning. However, there were differences in frequencies depending on the batch, thus, in autumn there was a higher frequency than expected for oestrus detected on day 4 (AR = 3.9, *p* = 0.003) and less on day 2 (AR =  − 3.3, *p* = 0.003). Eighty five percent of oestrus were detected at the 7:00 check, 7.8% at the 12:00 check and 6.3% at the 19:00 check. There was a difference in the duration of oestrus, being significantly longer in autumn and early spring and shorter in late spring and summer (*p* = 0.002). The length is shown in Fig. 1.

There was a negative partial correlation (controlled for parity and batch) between day of onset and length of oestrus (r = − 0.444, *p* = 0.002).

### Circadian rhythms

**Fig. 2:**
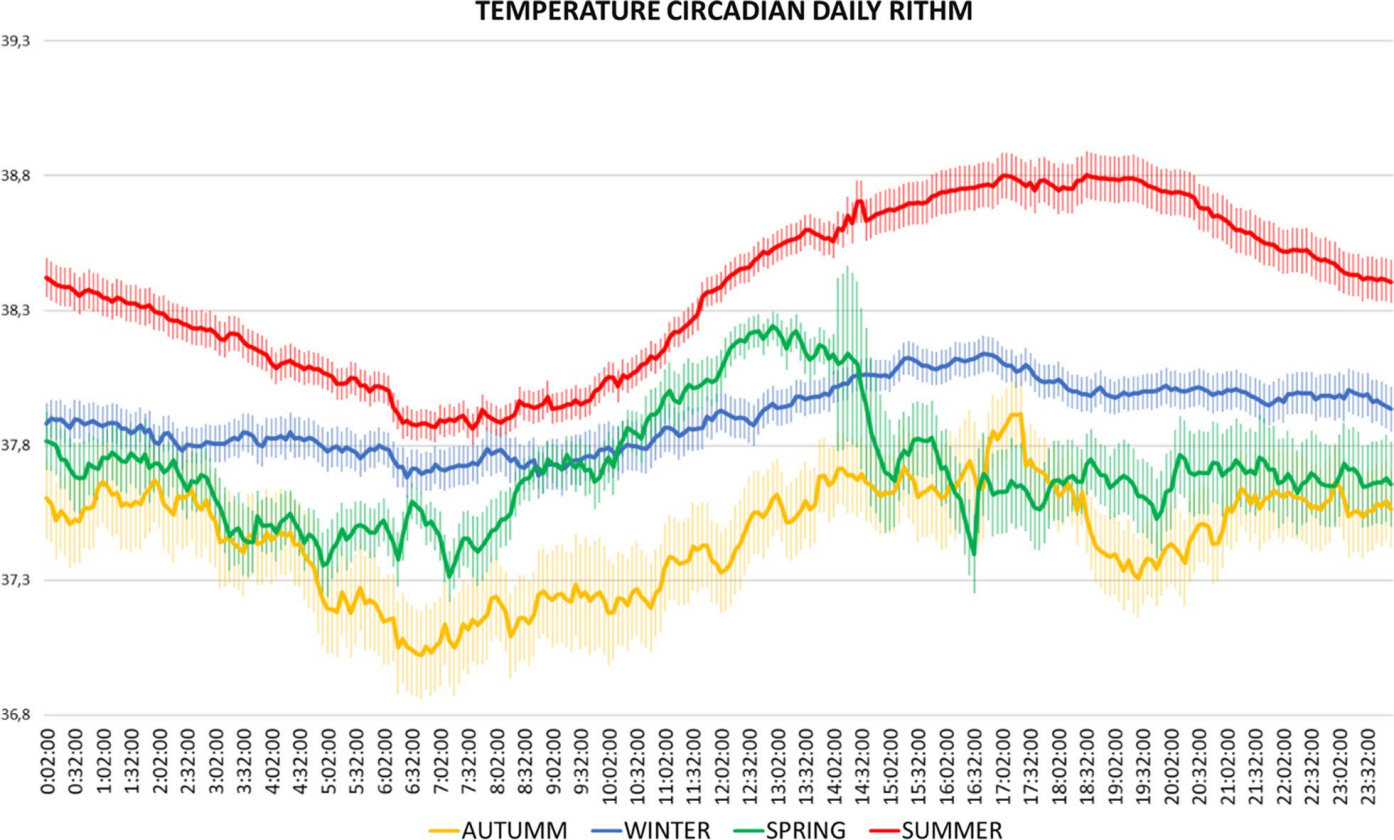
Mean line (± SEM) for core temperature interpolated with skin temperature in the four climatic seasons

The parameters related to the circadian rhythm of temperature are listed in Table 3. In addition, the average graph for temperature throughout the day at each station is shown in Fig. 2. According to the season of the year in which the temperature was taken; there is a clear difference between winter and spring with autumn and summer, with much less stability in the circadian rhythm in the latter two seasons. The same is true for temperature variability, which decreases significantly in summer and autumn. There were no differences in the time of day that represents the central point of the 8 consecutive hours with the highest and lowest temperature, but there were differences in the values of these temperatures, both in the minimum and in the maximum. However, while in the minimum the difference is of all seasons within winter, in the maximum there is a clear difference of summer and autumn with winter and summer with spring.

**Table 3:** Parameters for circadian temperature rhythm calculated for each climatic season

Season	IS	IV	RA	TL8	TM8	L8	M8	MEAN
Winter	0.39 ± 0.24^a^	0.07 ± 0.007^a^	0.006 ± 0.001^a,c^	8:07 ± 1:32	13:48 ± 1:3	37.63 ± 0.1^a^	38.08 ± 0.06^a^	37.86 ± 0.07^a^
Early spring	0.37 ± 0.05^a^	0.06 ± 0.02^a^	0.003 ± 0.00^b^	7:52 ± 1:26	10:09 ± 1:16	37.86 ± 0.09^b^	38.05 ± 0.1^a^	37.95 ± 0.1^a,b^
Late spring	0.50 ± 0.06^b^	0.08 ± 0.02^a,b^	0.006 ± 0.001^a^	6:19 ± 1:17	10:31 ± 2:20	38.01 ± 0.12^b^	38.49 ± 0.13^b^	38.26 ± 0.11
Summer	0.75 ± 0.04^c^	0.04 ± 0.01^b^	0.016 ± 0.001^c^	6:02 ± 0:40	13:09 ± 1:31	38.07 ± 0.1^b^	39.28 ± 0.14^b^	38.70 ± 0.11^b^
Autum	0.17 ± 0.02^a^	0.12 ± 0.02^a,b^	0.01 ± 0.001^a^	8:02 ± 0:06	16:41:15	37.77 ± 0.1^b^	38.44 ± 0.12^c^	38.12 ± 0.12^c^
*p*-value	< 0.0001	0.004	< 0.0001	NS	NS	< 0.0001	< 0.0001	< 0.0001

Where IS = Interdaily stability, IV = Intradayly variations, RA = rhythm amplitude, TM8 = time at the maximum temperature, TL8 = time at the lowest temperature, L8 = lowest temperature, M8 = maximum temperature, MEAN = average of the parameters

Different superscripts in the same column indicate significant differences

It is observed that in late spring, and, specially, summer sows have more stability and less abrupt changes than during the rest of the year, as the thermal jump at this time of the year is smaller. It should be noted that the minimum and maximum temperatures recorded in this season are almost one degree Celsius higher than in the other seasons, except for late spring that is 0.6 °C. There were no differences in any of the circadian parameters when comparing the different parities.

The data obtained after the cosinor analysis are shown in the table below (Table 4).

**Table 4:** Cosinor analysis sorted by batches

Batch	Period	Robustness	Mesor	Amplitude	Acrophase
Winter	26.02 ± 0.86	17.58 ± 2.23^a^	37.76 ± 0.07^a^	0.27 ± 0.04	14:07 ± 1:28^b^
Early spring	26.46 ± 0.76	6.21 ± 1.22^a^	37.90 ± 0.12^a^	0.09 ± 0.01	8:32 ± 1:21^c^
Late spring	24.37 ± 2.16	21.6 ± 6.18^a^	38.19 ± 0.15^a,b^	1.15 ± 0.92	16:39 ± 1:51^b^
Summer	24.67 ± 0.28	50.77 ± 4.64^b^	38.55 ± 0.11^b^	0.63 ± 0.05	17:45 ± 0:22^b^
Autumm	22.92 ± 1.33	8.15 ± 1.05^a^	37.85 ± 0.22^a^	0.21 ± 0.06	5:41 ± 1:29^a^
*p*-value	NS	< 0.0001	< 0.0001	NS	< 0.0001

Different superscripts in the same column indicate significant differences

In all controlled batches the period is about 24 h with no differences between periods of the year, but we did find significant differences in robustness; much higher in summer than in the other periods which again indicates that the rhythms are much more stable than in the rest of the year as the sows are more influenced by the ambient temperature. We also found differences in the mesor when comparing summer with all other periods except late spring.

The acrophase of body temperature only coincides with the time of ambient Tmax in winter, being deviated in the other batches between 3 and 7 h.

### Correlation among length of oestrus and day of onset with circadian parameters

**Fig. 3:**
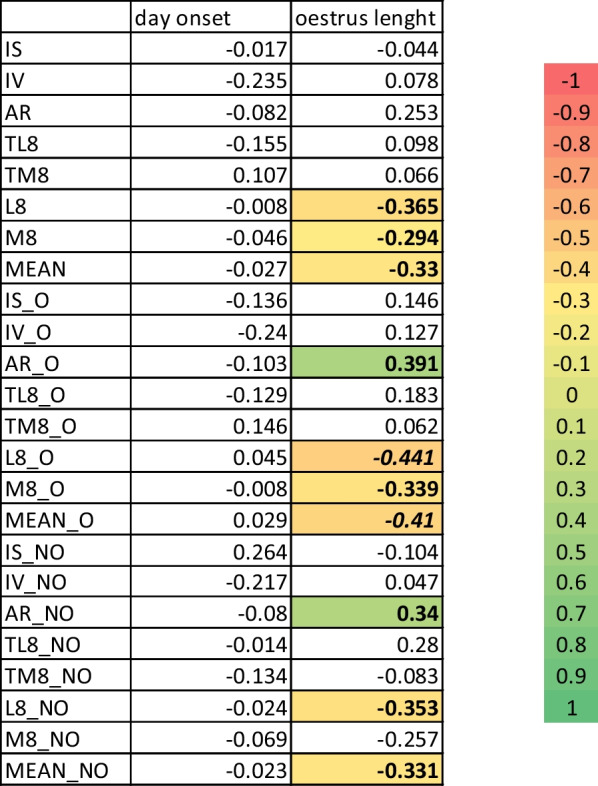
Heatmap for correlations among day of oestrus onset and duration with circadian parameters

Correlations between post-weaning day of oestrus onset and duration (in days) of oestrus are shown in Fig. 3.

There were no correlation between any of the parameters with the day of oestrus onset. However, most of the correlations found are negative. The observed correlations were among oestrus length and time for the lower or the higher temperature, and for the mean. Interestingly, the greater the amplitude (AR) both on the days without oestrus and on the days when the sows were in oestrus, the longer the oestrus lasted significantly.

### Comparison of parameters during oestrus anoestrus days

With regard to the circadian rhythm related parameters calculated during the days when the sows were in oestrus and the days when they were in anoestrus, differences in two parameters were observed, the IS (*p* = 0.044) with a mean of 0.670 ± 0.007 for the period of oestrus and 0.472 ± 0.032 for the period of oestrus, and for RA (*p* = 0.049) with 0.012 ± 0.002 for anoestrus period and 0.007 ± 0.001 for estrus days. The IS indicates stability in the circadian rhythm, with which the capacity of the sows to thermoregulate is measured (Table 5).

**Table 5:** Temperature circadian rhythm parameters for the period of oestrus and anoestrus

Parameter	Days	MEAN ± SEM	*P*-Value
IS	Oestrus	0.696 ± 0.029	NS
	Anoestrus	0.661 ± 0.034	
IV	Oestrus	0.096 ± 0.012	< 0.0001
	Anoestrus	0.057 ± 0.006	
RA	Oestrus	0.009 ± 0.001	NS
	Anoestrus	0.009 ± 0.001	
TL8	Oestrus	7:48:06 ± 0:24:29	0.003
	Anoestrus	10:03:24 ± 0:41:07	
TM8	Oestrus	12:01 ± 0:54	0.002
	Anoestrus	15:36 ± 0:32	
L8	Oestrus	37.55 ± 0.180	NS
	Anoestrus	37.58 ± 0.169	
M8	Oestrus	38.33 ± 0.22	NS
	Anoestrus	38.31 ± 0.17	
MEAN	Oestrus	37.98 ± 0.20	NS
	Anoestrus	37.97 ± 0.16	

Therefore, taking all data together, there are differences in IS, RA, TL8 and TM8 comparing the period when sows are in oestrus with the period when sows are not in oestrus. The same data were also analysed segmented by batches and the results are shown in Table 6.

**Table 6:** Temperature circadian rhythm parameters for the period of oestrus and anoestrus

Season			Media	*P*-value
Winter	IS	Oestrus	0,6300.08	NS
		No oestrus	0.50 ± 0.06	
	IV	Oestrus	0.07 ± 0.01	NS
		No oestrus	0.06 ± 0.01	
	RA	Oestrus	0.07 ± 0.002	NS
		No oestrus	0.01 ± 0.001	
	TL8	Oestrus	7:05 ± 0:58	0.006
		No oestrus	8:26 ± 0:29	
	TM8	Oestrus	17:30 ± 0:48:45	NS
		No oestrus	17:56 ± 0:51:26	
	L8	Oestrus	37.52 ± 0.19	NS
		No oestrus	37.55 ± 0.11	
	M8	Oestrus	38.07 ± 0.09	NS
		No oestrus	38.08 ± 0.08	
	MEAN	Oestrus	37.83 ± 0.12	NS
		No oestrus	37.84 ± 0.08	
Early spring	IS	Oestrus	0.59 ± 0.05	NS
		No oestrus	0.72 ± 0.05	
	IV	Oestrus	0.09 ± 0.02	NS
		No oestrus	0.05 ± 0.01	
	RA	Oestrus	0.002 ± 0.0005	NS
		No oestrus	0.003 ± 0.0005	
	TL8	Oestrus	7:29 ± 1:07	< 0.0001
		No oestrus	12:36 ± 2:02	
	TM8	Oestrus	13:40 ± 3:35	0.004
		No oestrus	10:37 ± 0:57	
	L8	Oestrus	37.88 ± 0.09	NS
		No oestrus	37.81 ± 0.11	
	M8	Oestrus	38.07 ± 0.1	NS
		No oestrus	38.03 ± 0.11	
	MEAN	Oestrus	37.98 ± 0.09	NS
		No oestrus	37.92 ± 0.11	
LATE SPRING	IS	Oestrus	0.62 ± 0.05	0.036
		No oestrus	0.77 ± 0.05	
	IV	Oestrus	0.15 ± 0.04	0.065
		No oestrus	0.08 ± 0.02	
	RA	Oestrus	0.0033 ± 0.002	0.035
		No oestrus	0.0078 ± 0.002	
	TL8	Oestrus	8:27 ± 1:28	NS
		No oestrus	9:51 ± 1:05	
	TM8	Oestrus	7:05 ± 0:57	< 0.0001
		No oestrus	17:31 ± 1:17	
	L8	Oestrus	38.03 ± 0.12	NS
		No oestrus	38.00 ± 0.12	
	M8	Oestrus	38.39 ± 0.12	0.013
		No oestrus	38.62 ± 0.18	
	MEAN	Oestrus	38.23 ± 0.12	0.044
		No oestrus	38.30 ± 0.13	
Summer	IS	Oestrus	0.87 ± 0.03	0.057
		No oestrus	0.77 ± 0.05	
	IV	Oestrus	0.051 ± 0.008	0.025
		No oestrus	0.032 ± 0.006	
	RA	Oestrus	0.017 ± 0.002	0.041
		No oestrus	0.014 ± 0.001	
	TL8	Oestrus	8:06 ± 0:10	NS
		No oestrus	7:51 ± 0:09	
	TM8	Oestrus	18:17 ± 0:27	0.003
		No oestrus	17:48 ± 0:16	
	L8	Oestrus	38.10 ± 0.12	0.031
		No oestrus	37.88 ± 0.14	
	M8	Oestrus	39.45 ± 0.21	0.006
		No oestrus	39.12 ± 0.16	
	MEAN	Oestrus	38.82 ± 0.16	0.045
		No oestrus	38.57 ± 0.13	
Autumm	IS	Oestrus	0.66 ± 0.11	0.053
		No oestrus	0.26 ± 0.04	
	IV	Oestrus	0.21 ± 0.04	0.03
		No oestrus	0.12 ± 0.02	
	RA	Oestrus	0.022 ± 0.005	0.034
		No oestrus	0.012 ± 0.004	
	TL8	Oestrus	7:43 ± 0:11	NS
		No oestrus	13:53 ± 3:29	
	TM8	Oestrus	17:37 ± 1:14	0.009
		No oestrus	11:37 ± 2:00	
	L8	Oestrus	34.19 ± 1.59	0.07
		No oestrus	35.35 ± 1.30	
	M8	Oestrus	35.81 ± 1.33	NS
		No oestrus	36.24 ± 1.15	
	MEAN	Oestrus	35.11 ± 1.40	NS
		No oestrus	35.76 ± 1.22	

While in the controlled heats in winter and early spring there is no difference in the circadian parameters of temperature, in autumn, late spring and summer differences or trends were found for IS, IV, RA, in spring for TL8, in summer for M8 and a tren for L8 and in autumm tren for L8 and M8. Interestingly, while IS and RA increase in summer and autumn, they decrease in spring and do not differ in winter. On the other hand, IV significantly increases in all seasons except winter, which does not vary.

The increase in IV, which is observed in all seasons except spring, is probably due to the effect of hormones that attempt to lower the temperature in the reproductive tract, since there is a detectable decrease of vaginal temperature during heat period [9].

The data obtained in the cosinor analysis both taking all data together and segmented by sampling period are shown in Tables 7 and 8.

**Table 7:** Cosinor normalized data during oestrus for each batch studied

TANDA	Period	Robustness	Mesor	Amplitude	Acrophase
Winter	24.926 ± 0.46	44.88 ± 5.57^a^	37.88 ± 0.07^a^	0.36 ± 0.06^a^	14:52 ± 1:26^a,b^
Early spring	25.16 ± 0.29	39.77 ± 6.07^a^	37.96 ± 0.09^a^	0.08 ± 0.01^b^	12:49 ± 1:27^b^
Late spring	24.77 ± 0.63	42.01 ± 9.83^a^	38.25 ± 0.13^a^	0.19 ± 0.03^a,b^	15:45 ± 2:00^a,b^
Summer	24.34 ± 0.24	79.01 ± 3.33^b^	38.80 ± 0.15^b^	0.81 ± 0.06^c^	18:43 ± 0:21^a^
Autumm	23.62 ± 0.89	31.04 ± 5.07^a^	37.59 ± 0.26^a^	0.30 ± 0.08^a,b^	18:11 ± 1:13^a,b^
*p*-value	NS	< 0.0001	< 0.0001	< 0.0001	0.011

Different superscripts in the same column indicate significant differences

**Table 8:** Cosinor normalized data comparing the whole recorded period and the oestrus period

Batch	Parameter	Period	Mean ± SEM	*P*-value
Winter	Period	Whole period	26.02 ± 0.86	NS
		Oestrus	24.92 ± 0.46	
	Robustness	Whole period	17.58 ± 2.23	< 0.0001
		Oestrus	44.88 ± 5.57	
	Mesor	Whole period	37.76 ± 0.07	NS
		Oestrus	37.88 ± 0.07	
	Amplitude	Whole period	0.27 ± 0.04	NS
		Oestrus	0.36 ± 0.06	
	Acrophase	Whole period	5:28 ± 1:28	NS
		Oestrus	2:43 ± 1:13	
Early spring	Period	Whole period	26.33 ± 0.81	NS
		Oestrus	25.16 ± 0.29	
	Robustness	Whole period	6.5 ± 1.28	< 0.0001
		Oestrus	39.77 ± 6.07	
	Mesor	Whole period	37.84 ± 0.12	NS
		Oestrus	37.96 ± 0.09	
	Amplitude	Whole period	0.08 ± 0.01	NS
		Oestrus	0.08 ± 0.01	
	Acrophase	Whole period	5:15 ± 1:27	NS
		Oestrus	5:14 ± 1:27	
Late spring	Period	Whole period	25.78 ± 1.83	NS
		Oestrus	24.77 ± 0.63	
	Robustness	Whole period	18.01 ± 5.62	NS
		Oestrus	42.01 ± 9.83	
	Mesor	Whole period	38.19 ± 0.17	NS
		Oestrus	38.25 ± 0.13	
	Amplitude	Whole period	1.23 ± 1.03	NS
		Oestrus	0.19 ± 0.03	
	Acrophase	Whole period	6:06 ± 2:02	NS
		Oestrus	6:00 ± 2:00	
Summer	Period	Whole period	24.57 ± 0.24	NS
		Oestrus	24.34 ± 0.24	
	Robustness	Whole period	53.63 ± 4.64	< 0.0001
		Oestrus	79.01 ± 3.33	
	Mesor	Whole period	38.58 ± 0.12	0.007
		Oestrus	38.80 ± 0.15	
	Amplitude	Whole period	0.69 ± 0.05	0.002
		Oestrus	0.81 ± 0.06	
	Acrophase	Whole period	1:38 ± 0:24	0.009
		Oestrus	1:25 ± 0:31	
Autumm	Period	Whole period	22.98 ± 1.63	NS
		Oestrus	23.62 ± 0.89	
	Robustness	Whole period	8.68 ± 1.12	0.017
		Oestrus	31.04 ± 5.10	
	Mesor	Whole period	37.80 ± 0.27	NS
		Oestrus	37.59 ± 0.26	
	Amplitude	Whole period	0.24 ± 0.06	a
		Oestrus	0.30 ± 0.08	
	Acrophase	Whole period	6:08 ± 1:44	0.003

There is no difference in the period when comparing the seven days recorded with the days when the sow is in oestrus. But there are differences in robustness in all periods studied; it increases significantly which means that the cycles become more stable. Mesor and amplitude increase in summer, mesor decreases in autumn and does not vary in the other periods. Likewise, amplitude increases in summer on the days of oestrus and does not vary in the other periods.

### Vaginal temperature

#### Variation during oestrus

The mean values obtained for the period before, after and during oestrus are shown in the Table 9 below.

**Table 9:** Values (Mean ± SEM) for the period studied

Season	Tvagbfr	Tvagoes	Tvagaft	ΔTvagbfr-Tvagoes
Winter	38.07 ± 0.12	37.72 ± 0.10^a^	37.83 ± 0.13	− 0.35
Early spring	38.16 ± 0.10	37.99 ± 0.10^a^	38.00 ± 0.19	− 0.17
Late spring	38.21 ± 0.17	38.02 ± 0.13^a^	37.96 ± 0.15	− 0.19
summer	38.42 ± 0.09	38.53 ± 0.07^b^	38.30 ± 0.18	0.11
autumm	38.05 ± 0.11	37.74 ± 0.06^a^	37.53 ± 0.18	− 0.31
*p*-value	NS	< 0.0001	NS	

Where Tvagbfr = vaginal temperature before oestrus; Tvagoes = vaginal temperature during oestrus; Tvagaft = vaginal temperature after oestrus, ΔTvagbfr-Tvagoes = difference between Tvagbfr and Tvagoes

Different superscripts in the same column indicate significant differences

Interestingly, there were no differences comparing the vaginal temperature before and after the oestrus period comparing the five batches recorded, but as regards the vaginal temperature during oestrus, there was a significant difference comparing the Tvag recorded during summer with all the other batches.

When Tvag during estrus was compared with Tvag before and after estrus, a significant difference was found between Tvagbfr and Tvgoes (*p* = 0.006), between Tvagbfr and Tvagaft (*p* = 0.017) but not between Tvagoes and Tvagaft (*p* = 0.596). The latter lack of difference could be explained by the fact that although the oestrus signals are no longer visible, the related hormonal events may continue. However, when segmented by each of the batches, the difference between Tvagbfr and Tvagoes is significant only in the winter batch (*p* = 0.002) while it has a trend in autumn (*p* = 0.068) and early spring (*p* = 0.066), but not in late spring or summer.

In all seasons except summer the vaginal temperature decreased during the period of estrus compared to the immediately preceding period. The difference between the mean temperature during the oestrus and the previous period was 0.25 °C, 0.19 °C, 0.12 °C and 0.52 °C for winter, spring, summer and autumn, respectively. Ordering this difference from highest to lowest, which corresponds to the order of the seasons starting with autumn, there is a linear decrease (r = 0.930, *p* = 0.07) of this difference.

Partial correlation controlled for parity and season showed a correlation between Tvag during oestrus with that before oestrus (r = 0.569, *p* < 0.001), and after oestrus (r = 0.622, *p* < 0.001).

Partial correlations controlled for batch and sow parity were calculated between vaginal temperatures and core temperatures calculated on the basis of skin temperature, obtaining positive correlations with Tvagwhole (r = 0.680, *p* < 0.001), Tvagoes (r = 0.644, *p* < 0.0001), Tvagbfr (r = 0.510, *p* < 0.0001) and Tvagaft (r = 0.466, *p* < 0.0001).

**Fig. 4:**
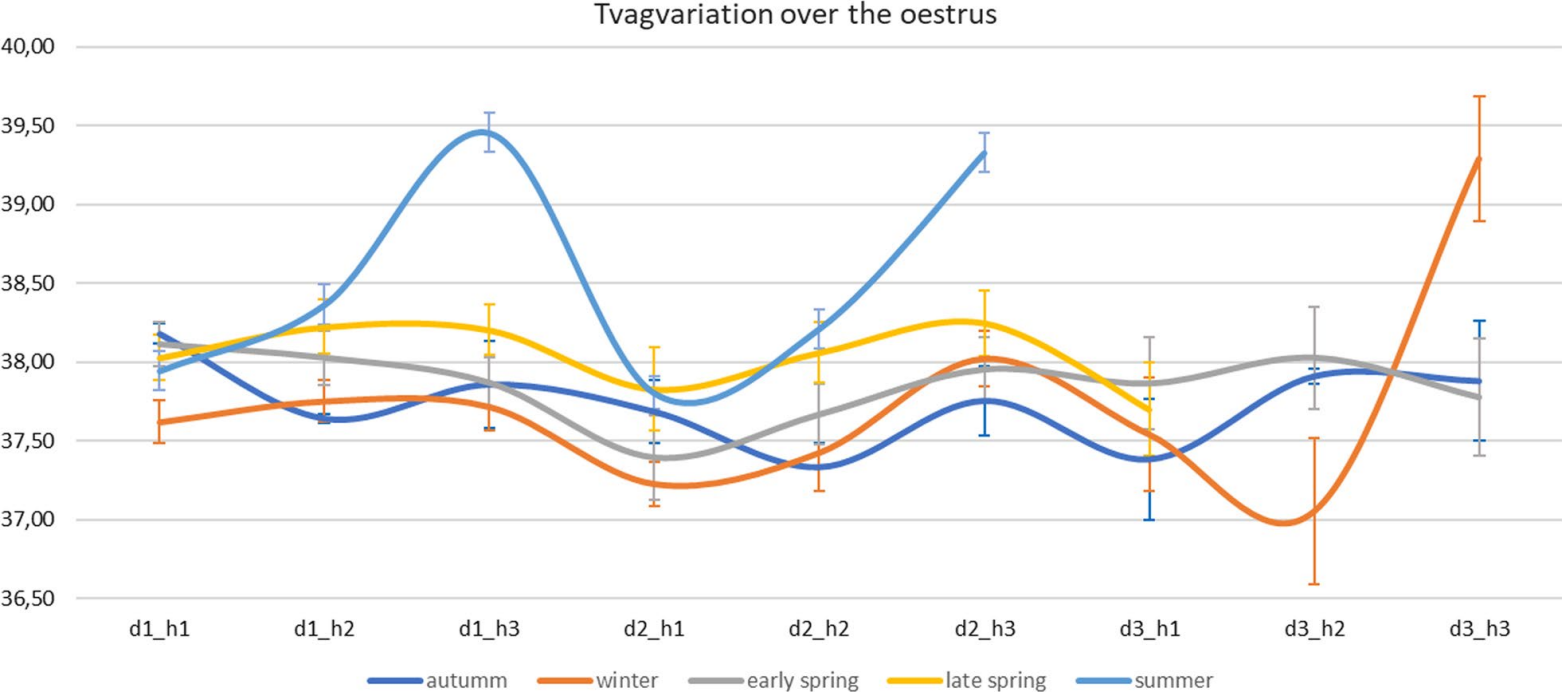
Evolution of vaginal temperature throughout the days of oestrus

Vaginal temperatures were compared for each of the measurements on the days the sows were in oestrus (Fig. 4), (Table 10).

**Table 10:** Cosinor analysis for Tvag during the oestrus sorted by batch

	Robustness	Mesor	Amplitude	Acrophase
Autumm	36.02 ± 5.65^a^	37.76 ± 0.08^a^	0.38 ± 0.072^a^	10:50 ± 3:46^a^
Winter	54.64 ± 8.07^a,b^	37.63 ± 0.11^a^	0.62 ± 0.12^a,b^	14:59 ± 1:20^a,b^
Early spring	46.33 ± 7.30^a^	37.77 ± 0.11^a^	0.43 ± 0.06^a^	9:46 ± 1:23^a^
Late spring	66.3 ± 7.91^b^	38.04 ± 0.20^a,b^	0.35 ± 0.07^a^	10:40 ± 2:08^a^
Summer	62.44 ± 3.10^b^	38.55 ± 0.10^b^	0.83 ± 0.08^b^	17:32 ± 0:25^b^
*p*-value	0.043	< 0.0001	0.001	< 0.0001

Different superscripts in the same column indicate significant differences

There appears to be a shortening of the temperature differences between the late spring and late summer measurements.

The cosinor analysis for Tvag indicated that, in robustness, there were differences between summer and late spring with autumn and early spring. In all other data, the difference was between summer and all other measurements except with late spring in mesor, with winter in amplitude and acrophase. It is interesting to see how the two batches with significantly higher Tmax and Tmed and lower Tmin and Tmed (environmental temperature) had the greatest amplitude in Tvag measured during estrus. This suggests that the amplitude of the circadian temperature rhythm at the vaginal level during oestrus is influenced by both high and low temperatures. It can also be seen that the acrophase of the Tvag is adjusted to the time of Tmax in winter and is delayed by about 3 h in summer, while in the other batches it is clearly ahead of the time of Tmax of the day. We can also observe that the acrophase of Tvag coincides with the acrophase of body temperature in winter and early spring, while it deviates in the other batches.

### Correlations among vaginal temperature and length of oestrus and day of onset

Controlled partial correlations were calculated for parity and season. A negative correlation was found between Tvag during oestrus with day of onset of oestrus (r = − 0.319, *p* = 0.015) and with duration of oestrus (r = − 0.363, *p* = 0.005) and while pre-oestrus temperature showed no correlation with either day of onset or duration, post-oestrus temperature showed a negative correlation with duration (r =  − 0.453, *p* < 0.001). Therefore, sows that started oestrus earlier had shorter oestrus duration and higher vaginal temperature during oestrus, and higher post oestrus temperature was correlated with shorter oestrus duration. However, controlling the correlations for ambient relative humidity, light and ambient temperature, the correlations between Tvag during oestrus and before oestrus (r = 0.533, *p* < 0.001) and after oestrus (r = 0.635, *p* < 0.001) and between Tvag after oestrus and duration of oestrus (r = − 0.300, *p* = 0.043) are maintained. The day of onset of oestrus and its duration are therefore influenced by temperature, humidity and luminosity, which are correlated with each other.

### Correlations among vaginal temperature and circadian parameters

**Fig. 5:**
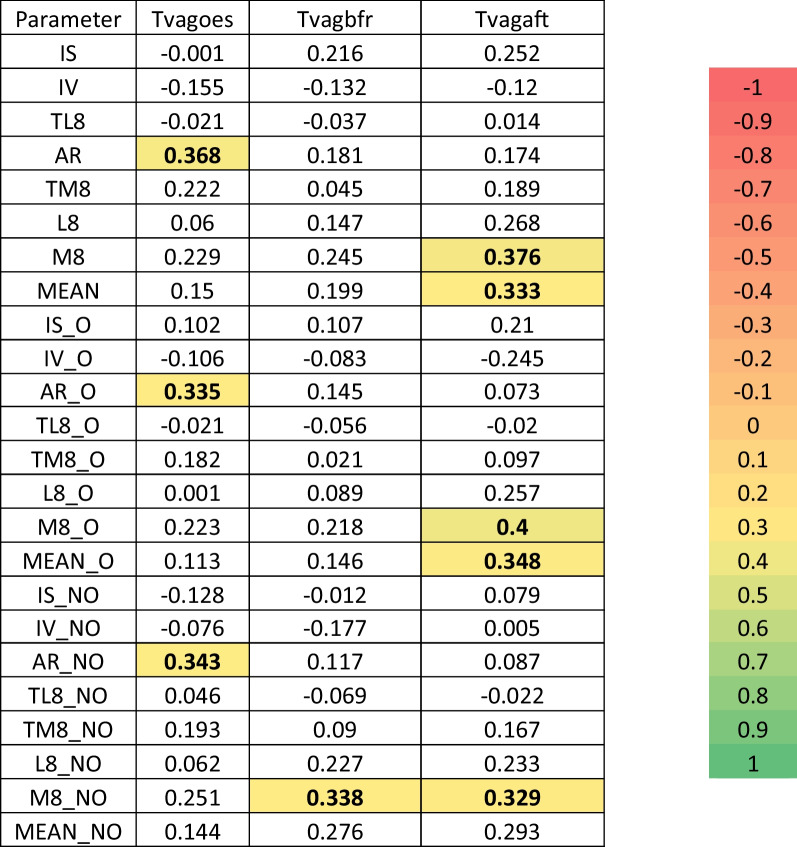
Correlations among vaginal temperatures and circadian parameters

Correlations between vaginal temperature before, during and after oestrus were calculated and are shown in Fig. 5.

### Correlation among vaginal temperature and environmental measures

The partial correlations controlled for batch and parity of the sow among vaginal temperature and environmental measurements showed positive correlation only with Luminosity and Tvagwhole (r = 0.373, *p* = 0.008) and Tvagoes (r = 0.328, *p* = 0.21).

### Correlation among Tvag and reproductive performances

The partial correlations showed positive correlation between Tvagoest and SBP_B (r = 0.242, *p* = 0.036), Tvagwhole and WTS_B (r = 0.220, *p* = 005), and Tvagaft with WTS_B (r = − 0.360, *p* = 0.004). Interestingly there was a negative correlation between Tvagaft and ABP_A (r = − 0.257, *p* = 0.049).

## Discussion

As far as we have been able to find in the literature, this is the first work to study the circadian cycle of sows during the post-weaning period until service. So far, much emphasis has been placed on the use of skin, rectal or vaginal temperature to determine sow [7, 10] or cow [14, 22] oestrus or ovulation timing, but the circadian parameters of this period have not been described.

Changes in the core temperature of the animals has been reported since decades [23–25] and it is well known the relation with physiology, well-being and productive performances, not only on the body temperature and its circadian behaviour but also on the environmental temperature cycles surrounding the animal [26]. Even body temperature has been proposed as a promising parameter for monitoring animal welfare or health status [27]. There is already ample evidence that in mammals the circadian rhythm is deeply related to metabolism [1].

The study of the circadian rhythm of temperature is easy in humans; with the simple use of a standard thermometer [1], serial measurements can be made. However, obtaining data over long periods of time requires other types of instrumentation. Similarly, serial measurements with clinical thermometers are not possible in animals because the data acquisition method itself is stressful and could lead to unwanted variations. This is why non-interactive, stand-alone acquisition systems have been developed, such as thermosensitive radio transmitters which, once ingested, emit continuous information about the internal temperature in the digestive tract. But again, these systems are suitable for humans where it is easy to retrieve the devices but not feasible in livestock such as pigs, where the floor is usually slatted to remove faeces. Therefore, in animals it is necessary, in most cases, to implant devices that read and record data and then retrieve them or emit them by readiofrequency. Among these are the iButtons (Maxim Integrated, San Jose, California) used in this experiment. These devices have been used in rats [28], mice, and in pigs have been validated by comparing cervical subcutaneous temperature with neck skin temperature [11].

On the other hand, it is important how the temperature of the animals is measured to obtain information and conclusions, as a significant part of the reported studies have been carried out under laboratory [27] or controlled conditions in terms of light, temperature or even nutrition. This study has been carried out in a conventional farm that shares conditions with many thousands of other farms around the world; and in subtropical climatic conditions that may condition the behavior of the temperature rhythm or reproductive performance.

There is no published information on IS, IV, L8, M8, TL8 or TM8 in pigs under any physiological circumstances. It is evident that the greatest changes when comparing temperature before, during and after oestrus occur in summer. The decrease in IV and the increase in TL8 and TM8 occur in summer but also in autumn. This may be related to weather conditions; there is no difference in mean Tmax in the controlled batches in autumn, winter and early spring, but there is in late spring and summer.

The mesor obtained from all animals was 38.12 °C, very close to the 38.5 °C recorded by Hanneman et al., [27] although these authors worked in laboratory conditions with non-breeding age animals (4.5–5 months old). Similarly, if we compare the amplitude recorded in that work it is much lower than that recorded in this work taking all data (0.18 vs. 0.48) but again the physiological state and controlled conditions of Hanneman's study make a substantial difference with commercial farm and breeding sow conditions. The fact that there is a significant difference in the robustness of the cycle when comparing periods when the sow is in oestrus with those when she is not, increasing during oestrus could be related to the hormonal release that induces oestrus and ovulation. The fact that in summer, a period in which there is a significant increase in anoestrus in different areas all over the world [29, 30], the robustness is significantly higher than in the other periods of the year could be the key to this observed seasonal reproductive alteration. In dairy cows a seasonal influence on the circadian rhythm of vaginal temperature has been demonstrated, with a flattening and lower amplitude in winter compared to spring and summer [31]. In our study a similar effect occurs; with an increased amplitude in summer, but also in winter with a flattening of the cycles in spring and autumn. However, in the sows studied, core temperature interpolated with skin temperature showed an increase in amplitude in late spring and summer compared to autumn, winter and early spring. In Kendall and Webster's study they find an acrophase advance of 5 h in autumn, while we find an advance of between 6 and 9 h in early spring and autumn compared to winter, summer and late spring. Once again we have to take into account that this trial was conducted in Hamilton, New Zealand, having a template clime in comparison with the dry Mediterranean clime of Murcia.

Age and physical exercise have been shown to interact with the circadian rhythm of temperature [2], but in this study no difference was observed due to the parity of the sows. To properly compare these results it must be taken into account that the sows included in the study did not exceed 9 farrowings, which is a chronological age of 4.5 years in animals that have a life expectancy of 15 years, although some authors claim that pigs have a similar life expectancy to humans [32]. And regarding physical exercise, the animals were monitored during the first week, in which the sows are housed in cages with restricted movement, so this factor is not a source of differences between individuals. In fact, in humans it has been described how activity and body temperature are coupled resonators, but with temperature taking the lead [33] case, as the animals are housed in cages (in accordance with the European welfare legislation contained in COUNCIL DIRECTIVE 2008/120/EC of 18 December 2008 laying down minimum standards for the protection of pigs [34], the physical activity-temperature interaction loses relevance.

Another factor influencing circadian temperature is feeding; in light-isolated and temperature-controlled animals, the main variation that occurs is due to feed intake [15]. In our study the sows are fed twice a day, but in addition they are subjected to variations in light, temperature and humidity [15]. In cows, it has been shown that intensive housing conditions with increased stocking density do not affect any of the cosinor parameters measured except for acrophase [12].

The decrease in vaginal temperature during oestrus coincides with the decrease in vulvar skin temperature measured by infrared [7]; there is no variation during proestrus but it clearly decreases at the onset of oestrus, as in our measurements. In this study we observed a decrease in Tvag during estrus. A drop in vulvar skin temperature of up to 1.5 °C at the onset of estrus has been observed previously [6] In this study the maximum drop observed was 0.31 °C in autumn in vaginal temperature, and a decrease in M8 for summer of 0.33 °C. It should be noted that Scolari’s study was carried out on animals housed in temperature and humidity controlled facilities, not subject to variations as in the present study, where it was intended to observe the influence of climate. In fact, in the present study there is a clear variation in the difference in vaginal temperature observed before and during oestrus, ranging from 0.31 °C in autumn to 0.11 °C observed in summer. On the other hand, in cows, Wang et al. [22] found greater variation in Tvag before and after oestrus onset in summer than in autumn or winter, contrary to what we found in this study. It should be taken into account, in addition to the differences that may result from the fact that they are different species, that this study was conducted in Shijiazhuang (China), with a much milder climate than in SE Spain. In France, the decrease in vulvar temperature related to the onset of oestrus has already been documented by Simoes et al. [7] but using infrared thermography. Our data and Simões ones are in clear disagreement with the finding of Soede et al. [35] who find no relationship between vaginal temperature and ovulation. In our findings it is striking that in summer there is no decrease in vaginal temperature, but an average increase of 0.11 °C. If vaginal cooling has implications for reproduction, in summer the controlled sows fail to reduce this temperature which could have consequences for reproductive efficiency. It has already been shown that high temperature environments produce alterations in the temperature rhythm and in the increase in body temperature during oestrus [36]. In cows it has been shown that ambient temperature and humidity have more influence on Tvag than on rectal temperature [37].

As regards day of oestrus onset and duration of oestrus, in cows there was no significant difference in the duration of estrous between summer and winter [38].

## Conclusions

There is a seasonal influence on the circadian rhythm of temperature and summer is clearly the season with the greatest differences in circadian parameters when compared to the other seasons. Not only that, but the largest differences in body temperature and vaginal temperature comparing oestrus and non-oestrus periods also occur in this season and in late spring.

Physiologically there are variations in the temperature rhythm that are influenced by the climate. In this study, the extreme summer conditions seem to definitely influence this rhythm and make the body and vaginal temperature of the sows different from the rest of the year.

The increase in period robustness in both body and vaginal temperature during the days when sows are in oestrus could be related to the hormonal events of oestrus and ovulation and seems to be independent of weather since it occurs in all controlled seasons. This robustness is significantly higher in summer than in the other seasons both in the oestrus period and on days when sows are not in oestrus. This could be due to the weather, as the daily temperature variation is lower in summer than in the other seasons.

## Data Availability

Data are available at request to the authors.
